# Suvmax of the lesion should be considered in the treatment plan for stage I non-small cell lung cancer

**DOI:** 10.1007/s12149-025-02049-0

**Published:** 2025-04-10

**Authors:** Gökhan Kocaman, Farrukh Ibrahımov, Yusuf Kahya, Mine Araz, Atilla Halil Elhan, Serkan Enön

**Affiliations:** 1 School of Medicine Thoracic Surgery Department, Ankara University Medicine Faculty İbn-I Sina Hospital, Ankara University, 06100 Sıhhiye/Ankara, Turkey; 2 School of Medicine Nuclear Medicine Department, Ankara University Medicine Faculty, Ankara University, 06100 Sıhhiye/Ankara, Turkey; 3 School of Medicine Biostatistics Department, Ankara University Medicine Faculty, Ankara University, 06100 Sıhhiye/Ankara, Turkey

**Keywords:** Non-small cell lung cancer, SUVmax, Prognosis, Sublobar resection

## Abstract

**Objectives:**

High maximum standardized uptake value (SUVmax) is associated with poorly differentiated tumors and lymph node metastasis. It is still controversial which tumors can be treated with sublobar resection and there are publications stating that SUVmax of the tumor may be important in choosing sublobar resection. Our aim in this study is to examine the prognostic value of tumor SUVmax in stage 1 non-small cell lung cancer and to determine its place in sublobar resection preference.

**Methods:**

The study included 314 patients who underwent wedge resection, segmentectomy or lobectomy for pathological stage I NSCLC with tumor size ≤ 3 cm between January 2008 and December 2020. SUVmax of the tumors are dichotomized according to ROC threshold value 5.2 and prognostic factors for recurrence-free and overall survival were analysed.

**Results:**

In the multivariate survival analysis, SUVmax (p = 0.012), lymphovascular and/or perineural invasion (p < 0.001) and visceral pleural invasion (p = 0.031) were found to be independent prognostic factors for recurrence-free survival; age (p = 0.027), sex (p = 0.010) and SUVmax (p = 0.036) for overall survival. While there was no difference between lobar or sublobar resection in terms of recurrence-free survival (p = 0.647) in patients with SUVmax ≤ 5.2, lobectomy was found to be advantageous over sublobar resection for recurrence-free survival in patients with SUVmax > 5.2 (76.6% ± 3.9% / 53.4% ± 12.1%, p = 0.006, respectively).

**Conclusions:**

High SUVmax (> 5.2) is associated with poor recurrence-free survival and overall survival rates in pathological stage 1 NSCLC patients. In stage 1 patients, sublobar resection should be avoided if the primary tumor has a high SUVmax.

## Introduction

According to the results of a randomized controlled study comparing lobectomy and sublobar resection in T1N0 non-small cell lung cancer, published by the lung cancer study group in 1995, local recurrence 3 times and cancer related deaths were observed 2 times more frequently in patients who underwent sublobar resection [1]. Due to the results of this study, lobectomy has been accepted as the standard treatment method in T1N0 NSCLC patients and sublobar resections were preferred for only selected patients with compromised respiratory capacity. Nowadays, with the frequent use of computed tomography, the detection rate of small-diameter, subsolid nodules has increased. This situation has led to the more intensive use of sublobar resections in small peripheral tumors that are normally suitable for lobectomy. The term sublobar resection includes two different types of resection; wedge resection and segmentectomy. While segmentectomy is an anatomical resection that includes hilar lymph node dissection, wedge resection is a non-anatomical resection in which only the targeted tumor tissue is removed. The JCOG0802/WJOG4607L [2] study published in 2022 showed that segmentectomy provided overall survival results equivalent to lobectomy in peripheral tumors with a diameter of ≤ 2 cm, and the CALGB 140503 [3] study published in 2023 showed that disease-free survival results equivalent to lobectomy could be achieved with sublobar resections in patients with ≤ 2 cm tumors without lymph node metastases. Despite all these developments, it is still controversial which tumors can be treated with sublobar resection. FDG-PET is now routinely used for staging purposes in NSCLC and high maximum standardized uptake value (SUVmax) is associated with poorly differentiated tumors and lymph node metastasis [4]. There are publications stating that SUVmax of the tumor may be important in choosing sublobar resection as tumors with a high SUVmax are prone to recurrence and sublobar resections should be avoided for these tumors [5, 6].

Our aim in this study is to examine the prognostic value of tumor SUVmax in Stage 1 NSCLC and to determine its place in sublobar resection preference.

## Materials and methods

The study included 314 patients who underwent wedge resection, segmentectomy or lobectomy for pathological stage I NSCLC with tumor size ≤ 3 cm between January 2008 and December 2020. Patients with neoadjuvant therapy, central tumors or tumors larger than 3 cm in longest diameter, patients who underwent bilobectomy or pneumonectomy, R1–R2 resections, carcinoid tumors, or synchronous tumors were excluded from the study. Patients were staged according to the 8th TNM classification. Thorax computed tomography (CT), cranial CT/ magnetic resonance imaging (MRI), positron emission tomography-CT (PET/CT) were used for clinical staging. Endobronchial ultrasound-guided transbronchial needle aspiration (EBUS-TBNA) or mediastinoscopy were used for invasive mediastinal staging, when necessary.

Patients fasted for at least 6 h, with blood glucose levels confirmed to be below 200 mg/dL before PET-CT imaging. Following intravenous injection of about 370 MBq 18F-FDG, whole body PET/CT acquisition was started after an interval of 60 min. A non-contrast CT scan (120 kV, 70 mA, tube rotation time of 0.5 s per rotation, a pitch of 1.375, and a slice thickness of 3.3 mm) was performed for attenuation correction and anatomic correlation of the PET images obtained by a PET scan of 2 min/bed position, covering the area from the vertex to the proximal femur. Semiautomated volume of interests (VOI) were drawn around target lesions with a treshold SUVmax of 40% to define contours. SUVmax calculations were then made for the primary lung lesion.

The patients were evaluated in the multidisciplinary thoracic oncology council for the treatment plan. Sublobar resections were generally preferred for patients with limited respiratory reserve and high comorbidity or subsolid nodules smaller than 2 cm, depending on the preference of the principle surgeon. The patients were followed up with thorax CT every 6 months for 5 years, and then annually for at least 10 years. Local recurrence refers to staple line, bronchial stump, hilar or ipsilateral mediastinal lymph node and pleural recurrence. Systemic recurrence refers to lymph node, lung and distant organ recurrences other than local recurrence sites.

Descriptive data were presented in mean ± standard deviation (SD), median (interquartile range) (min–max) or number and frequency where applicable. Difference between two groups for normally distributed continuous variables was evaluated by Student’s *t* test. Mann–Whitney *U* test was used to compare two groups in terms of ordinal or non-normally distributed continuous variables. Receiver operating characteristic (ROC) curves were used to describe the diagnostic performance of the variables of interest. Overall survival (OS) was determined as the time from surgery until death from any cause or last follow-up. Recurrence-free survival (RFS) was determined as the time from surgery until relapse or until the last follow-up period. The survival estimations were performed using the method of Kaplan–Meier algorithm, and the comparison between groups was evaluated with Log-rank test. Multiple Cox proportional hazard model was used to determine independent predictors of an outcome after adjustment for other explanatory variables. Variables with a *p*-value of less than 0.25 in the univariable Cox proportional hazards regression were selected as candidates for the multivariable model along with all variables of known clinical importance using purposeful selection method. The hazard ratio (HR) and the associated 95% confidence interval (CI) were calculated. Statistical analysis was performed using the IBM SPSS version 30.0 software (IBM Corp. Armonk, NY, USA). A two tailed p-value less than 0.05 was considered statistically significant.

## Results

There were 95 (30.3%) female and 219 (69.7%) male patients. The median age of the patients was 62 years (19–80, IQR:12). While 72 (22.9%) of the patients had never smoked, 242 (77.1%) patients were active or former smokers. Median SUVmax was 5.4 (0–32, IQR:7.9). Median tumor diameter was 19.5 mm (3–30 mm, IQR:12). 60 (19.1%) patients underwent wedge resection, 14 (4.5%) patients underwent segmentectomy, and 240 (76.4%) patients underwent lobectomy. 219 (69.7%) patients had adenocarcinoma, 67 (21.3%) patients had squamous cell carcinoma, and 28 (9%) patients had other (pleomorphic, large cell carcinoma, adenosquamous cell carcinoma) histopathological type tumors. Lymphovascular and/or perineural invasion was detected in 39 (12.4%) patients. Stage distribution of the patients was as follow; stage 1A1: 42 (13.4%) patients, 1A2: 109 (34.7%) patients, 1A3: 82 (26.1%) patients, 1B: 81 (25.8%) patients. Visceral pleural invasion was present in 81 (25.8%) patients. 14 (4.5%) patients received adjuvant chemotherapy. Recurrence was observed in 56 (17.8%) patients (16 local, 26 systemic and 14 local + systemic recurrences) and 47 (15%) patients died during follow-up period.

In the ROC analysis for SUVmax of the tumor in terms of recurrence, the value of 5.2 was chosen as the threshold value. ((AUC = 0.599 ± 0.038 (95% CI: 0.525–0.673), p = 0.090, sensitivity = 0.714 (95% CI: 0.585–0.816), specificity = 0.516 (95% CI: 0.455–0.576)). Same SUVmax threshold value (5.2) was chosen in the ROC analysis for survival (AUC:0.625 ± 0.040 (95%CI:0.546–0.703), p = 0.020). When the patients were analyzed by dividing them into two groups according to the SUVmax threshold value of 5.2, it was observed that there was a significant difference between the groups in terms of sex distribution, tumor diameter, smoking status, resection type, histological type, lymphovascular and/or perineural invasion status, stage distribution, recurrence and survival status (Table 1).

**Table 1:** Patient characteristics according to SUVmax

Characteristics n (%)	SUVmax ≤ 5.2 153 (48.7)	SUVmax > 5.2 161 (51.3)	P
**Sex**			** < 0.001**
Female	65 (42.5)	30 (18.6)	
Male	88 (57.5)	131 (81.4)	
**Age** (median, IQR)	61 (13)	63 (10)	0.116
**Tumor diameter** (median, IQR)	15 (10)	21 (8)	** < 0.001**
**Smoking status**			**0.008**
Former/current	108 (70.6)	134 (83.2)	
Never	45 (29.4)	27 (16.8)	
**Resection**			** < 0.001**
Sublobar resection	51 (33.3)	23 (14.3)	
Lobectomy	102 (66.7)	138 (85.7)	
**Histology**			** < 0.001**
Adenocarcinoma	131 (85.6)	88 (54.7)	
Non-adenocarcinoma	22 (14.4)	73 (45.3)	
**Lymphovascular and/or perineural invasion**			**0.017**
Present	12 (7.8)	27 (16.8)	
Absent	141 (92.2)	134 (83.2)	
**Visceral pleural invasion**			0.158
Present	34 (22.2)	47 (29.2)	
Absent	119 (77.8)	114 (70.8)	
**Pathological stage**			** < 0.001**
IA1	39 (25.5)	3 (1.9)	
IA2	56 (36.6)	53 (32.9)	
IA3	24 (15.7)	58 (36)	
IB	34 (22.2)	47 (29.2)	
**Adjuvant chemotherapy**			0.123
Yes	4 (2.6)	10 (6.2)	
No	149 (97.4)	151 (93.8)	
**Recurrence**			**0.002**
Yes	17 (11.1)	39 (24.2)	
No	136 (88.9)	122 (75.8)	
**Survival status**			**0.002**
Exitus	13 (8.5)	34 (21.1)	
Alive	140 (91.5)	127 (78.9)	

The median follow-up period of the patients was 68.5 months (1–194 months, IQR:41). The 5-year recurrence-free survival rate of patients was 80.4% ± 2.5% (median not reached). The 5-year overall survival rate was 87.4% ± 2% (median not reached).

In the analysis for 5-year recurrence-free survival, there were no differences between sexes (p = 0.114), smoking status (never/current or former, p = 0.475), lobar /sulobar resection types (p = 0.167), adenocarcinoma/non-adenocarcinoma histological types (p = 0.315), and in terms of adjuvant chemotherapy receiving status (p = 0.623). Statistically significant survival difference was observed between visceral pleural invasion status (absent /present; 84.1% ± 2.6% / 69.8% ± 5.6%, p = 0.018), lymphovascular and/or perineural invasion status (absent / present; 84.1% ± 2.4%/ 51.4% ± 9.5%, p < 0.001) and SUVmax (≤ 5.2 / > 5.2; 87.3% ± 3% / 73.6% ± 3.8%, p = 0.001).

In the analysis for 5-year overall survival, no difference was observed in terms of lobar / sublobar resection (p = 0.990), visceral pleural invasion status (p = 0.321), adenocarcinoma /non-adeno carcinoma (p = 0.943), receiving adjuvant chemotherapy (p = 0.354). Statistically significant survival difference was observed between sexes (f/m: 95.3% ± 2.3%/ 83.7% ± 2.7%, p < 0.001), smoking status (never/ current or former; 91.9% ± 3.5% / 86% ± 2.4%, p = 0.04), lymphovascular and/or perineural invasion status (absent / present; 89.3% ± 2% / 71.2% ± 8.6%, p = 0.019) and SUVmax (≤ 5.2 / > 5.2; 92% ± 2.3% / 83% ± 3.1%, p = 0.001).

In the multivariate survival analysis, SUVmax (p = 0.012), lymphovascular and/or perineural invasion (p < 0.001) and visceral pleural invasion (p = 0.031) were found to be independent prognostic factors for recurrence-free survival (Table 2); age (p = 0.027), sex (p = 0.010) and SUVmax (p = 0.036) for overall survival (Table 3).

**Table 2:** Cox regression analysis for recurrence-free survival

Variables	Univariable		Multivariable	
	HR (95%CI)	p	HR (95%CI)	p
Age	1.027 (0.995–1.061)	0.103		
Male vs female	1.641 (0.882–3.053)	0.118		
Current or former smoker vs never smoker	0.810 (0.453–1.447)	0.476		
Tumor diameter	1.025 (0.985–1.066)	0.218		
SUVmax > 5.2 vs ≤ 5.2	2.488 (1.407–4.400)	**0.002**	2.106 (1.180–3.760)	**0.012**
Sublobar resection vs lobectomy	1.492 (0.842–2.644)	0.170		
Adenocarcinoma vs non-adenocarcinoma	1.361 (0.743–2.493)	0.318		
Lymphvascular or perineural invasion present vs absent	3.681 (2.024–6.697)	** < 0.001**	3.124 (1.700–5.740)	** < 0.001**
Visceral pleural invasion present vs absent	1.892 (1.106–3.237)	**0.020**	1.806 (1.054–3.095)	**0.031**
Adjuvant chemotherapy present vs absent	1.337 (0.417–4.284)	0.625

**Table 3:** Cox regression analysis for overall survival

Variables	Univariable		Multivariable	
	HR (95%CI)	p	HR (95%CI)	p
Age	1.052 (1.014–1.091)	**0.006**	1.043 (1.005–1.082)	**0.027**
Male vs female	5.036 (1.807–14.035)	**0.002**	3.876 (1.373–10.937)	**0.010**
Current or former smoker vs never smoker	2.546 (1.007–6.437)	**0.048**		
Tumor diameter	1.039 (0.995–1.085)	0.085		
SUVmax > 5.2 vs ≤ 5.2	2.716 (1.433–5.150)	**0.002**	2.002 (1.046–3.833)	**0.036**
Sublobar resection vs lobectomy	0.996 (0.507–1.956)	0.990		
Adenocarcinoma vs non-adenocarcinoma	1.023 (0.547–1.912)	0.943		
Lymphvascular or perineural invasion present vs absent	2.345 (1.124–4.892)	**0.023**		
Visceral pleural invasion present vs absent	1.363 (0.738–2.517)	0.323		
Adjuvant chemotherapy present vs absent	1.729 (0.535–5.588)	0.360		

**Fig. 1:**
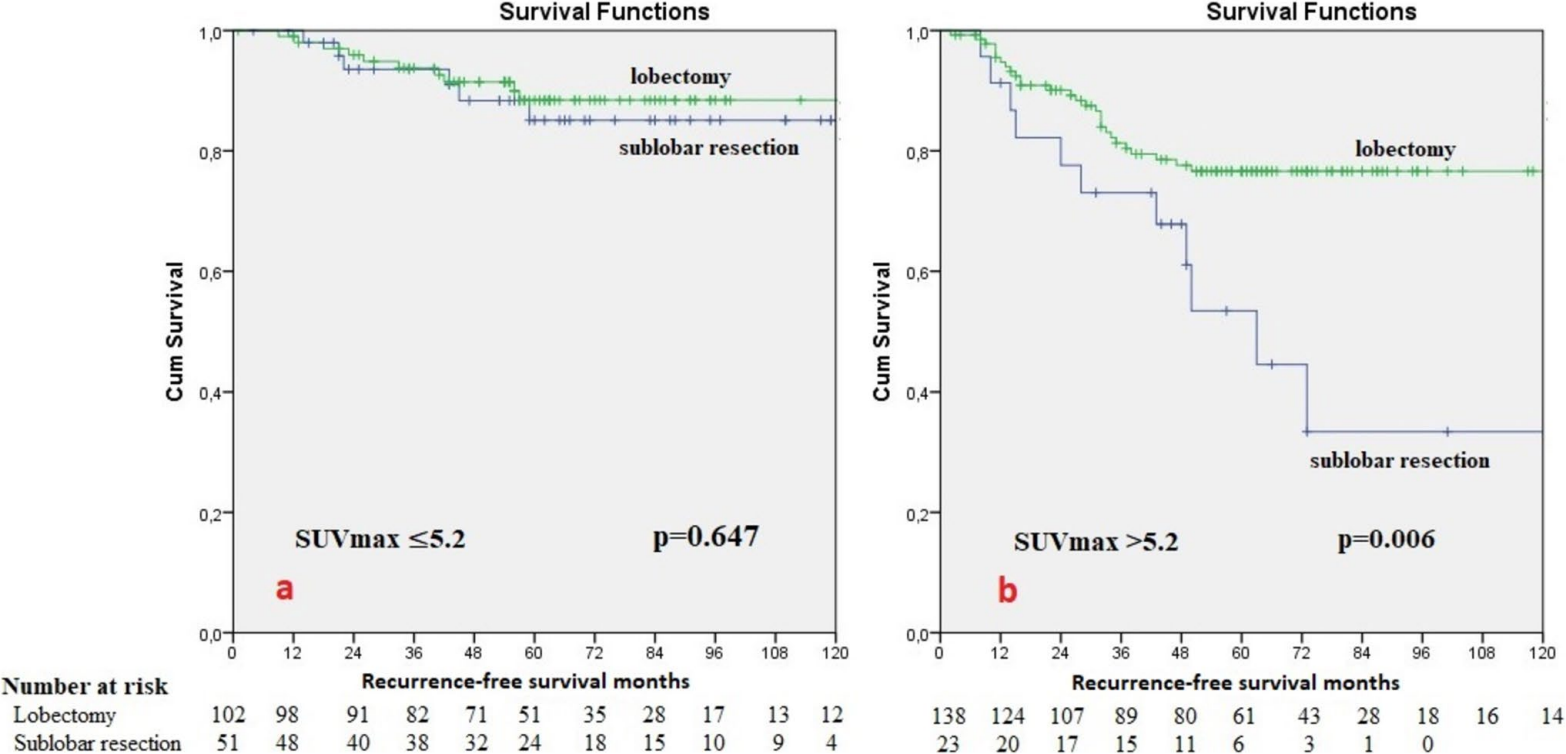
Recurrence-free survival analysis according to resection types for patients with SUVmax ≤ 5.2 (**a**) and SUVmax > 5.2 (**b**)

While there was no difference between lobar or sublobar resection in terms of recurrence-free survival (p = 0.647) in patients with SUVmax ≤ 5.2, lobectomy was found to be advantageous over sublobar resection for recurrence-free survival in patients with SUVmax > 5.2 (76.6% ± 3.9% / 53.4% ± 12.1%, p = 0.006, respectively) (Fig. 1).

## Discussion

SUVmax is the most commonly used PET parameter to express radiopharmaceutical uptake intensity. Specifically for 18F-FDG, intensity of uptake also is related with the biological behavior of the tumor. Molecular mechanisms are not fully explained but glucose transporter protein (GLUT1) expression and hexokinase activity are upregulated in NSCLC [7, 8]. High SUVmax shows the increased glucose avidity of the tumor due to the accelerated metabolism. High metabolic rate is related with high recurrence rates and poor outcomes [9, 10]. In our pathological N0, ≤ 3 cm NSCLC cohort, we found that higher SUVmax was associated with larger tumor diameter, higher smoking rate, more non-adenocarcinoma histology, lymphovascular and/or perineural invasion, recurrence, and death. SUVmax threshold value of 5.2 was an independent prognostic factor for both recurrence-free survival and overall survival. We also found that patients with SUVmax over 5.2 who underwent sublobar resection had shorter recurrence-free survival rate than those who underwent lobectomy. Although our patients are pN0, micrometastasis to the locoregional lymphatics and systemic micrometastasis that represents minimal residual disease is the possible explanation for higher recurrence rate in SUVmax > 5.2 group. Resection type (lobar/sublobar) changes the resection width including resected local lymphatics and lymph nodes so possibly affects the minimal residual disease. In their study, Bayarri-Lara et al. [11] showed the relation between SUVmax of the tumor and the presence of circulating tumor cells one month after the radical resection for NSCLC.

High recurrence rates in stage 1 NSCLC indicate that TNM staging alone is not sufficient to determine the prognosis of patients. Factors thought to be associated with high recurrence risk can be listed as increasing tumor diameter, poor differentiation, high grade, lymphovascular invasion, visceral pleural invasion, and spread through air spaces (STAS) positivity [12, 13]. It is extremely important that high SUVmax affects both recurrence-free and overall survival in stage 1 NSCLC, independent of known prognostic factors as in our study [4, 7, 8, 12, 14–16]. When found together with other risk factors, high SUVmax may constitute an indication for adjuvant treatment in stage 1 patients, even if they have a tumor of ≤ 3 cm. In addition, with more frequent and longer follow-up programs in these patients, possible relapses can be detected earlier and in case of recurrence, patients can be offered the chance of curative treatment.

PET/CT is frequently used in lung cancer staging today and SUVmax is routinely reported. The most important feature that distinguishes SUVmax from pathological prognostic factors is that it can be obtained relatively easily and in a standard way in the preoperative period. SUVmax may also play an important role in the preoperative treatment plan such as deciding the resection width. In their study published in 2024, Shiono et al. [5] examined the results of 723 clinical stage 1A lung cancer patients with a SUVmax ≥ 3 who underwent lobectomy or sublobar resection. Both overall survival and disease-free survival were worse in patients who underwent sublobar resection before and after propensity score matching (before matching: p < 0.001, p < 0.001; after matching: p = 0.008, p = 0.012, respectively) On the contrary, in their study published in 2020, Kamigaichi et al. [6] examined the results of 522 clinical stage 1A1-2 radiologically aggressive (consolidation/tumor ratio ≥ 0.8 and SUVmax ≥ 2.5) lung cancer patients. In both pre-matching and post-matching analyses, no survival difference was observed between patients who underwent lobectomy or segmentectomy in terms of 5-year recurrence-free survival and overall survival. The reason for the difference between the two studies may be the patient selection criteria. While in the first study, tumors with a diameter of up to 3 cm were included in the study; in the second study solid tumor diameter of less than 2 cm were included. In addition, while patients who underwent wedge resection were included in the first study, they were not included in the second study. In our study, similar to the first study, it was observed that patients with high SUVmax who underwent sublobar resection had worse recurrence-free survival rates than patients who underwent lobectomy. We found no relation between the surgical method and overall survival in the whole group or SUVmax ≤ 5.2 and SUVmax > 5.2 groups. Our cohort includes only stage 1 patients with long life expectancy (median age 62 years). Effective treatment strategies for recurrent tumors and low death rate in the study period (15%) possibly percludes the relation between the surgical method and overall survival.

There are many studies examining PET parameters in lung cancer from a prognostic perspective. While SUVmax is the mostly used parameter throughout these studies, a wide range of threshold values (2.5–20) are given for differantiating good and bad prognosis [4, 7, 9, 10, 17–21]. Differences in the stage and histological features of the patient group; imaging technique, device and software dissimilarities included in the studies may explain the wide range specified for the SUVmax threshold value. It does not seem possible to find a universal threshold for SUVmax. It is known that lower SUVmax is measured in adenocarcinomas than in squamous cell carcinomas [7, 17].Tumor size and ground glass ratio can also affect SUVmax [8, 18, 22, 23]. Instead, a different approach may be required for each scenario. While an average threshold value (such as SUVmax 5) can be used in patients with unknown preoperative diagnosis, as in our patient group; lower threshold values can be used in patients with a ground-glass appearance or when preoperative adenocarcinoma diagnosis is considered. These values can also be revised according to the tumor diameter. In addition, it may be more accurate for centers to obtain threshold values based on their own analysis results, taking into account the PET devices, protocols and patient populations. In this way, patient-based high/low SUVmax groupings can be done and this grouping may be more effective in guiding treatment.

This is a single center retrospective study with inevitable patient and treatment selection biases and limitations. As discussed earlier the results of SUVmax analyses can’t be generalized easily.

In conclusion, high SUVmax (> 5.2) is associated with poor recurrence-free survival and overall survival rates in pathological stage 1 NSCLC patients. We also found that patients with SUVmax over 5.2 who underwent sublobar resection had shorter recurrence-free survival rate than those who underwent lobectomy. So, sublobar resection should be avoided if the primary tumor has a high SUVmax.

## Data Availability

The data underlying this article will be shared on reasonable request to the corresponding author.
